# The Underpinnings of Ageism: Multiple Mediational Model of Epistemological Style, Social Dominance Orientation, Right-Wing Authoritarianism, and Ageist Attitudes

**DOI:** 10.1155/2019/3672725

**Published:** 2019-11-03

**Authors:** Richard S. Henry, Paul B. Perrin, Erin R. Smith

**Affiliations:** Virginia Commonwealth University, Richmond, VA, USA

## Abstract

This study seeks to understand the psychological factors that may contribute to the development and endorsement of ageist belief systems. Dual process theory is used to examine how one's worldview, beliefs in social hierarchy, authoritarian aggression, authoritarian submission, and conventionalism predict ageist attitudes. Participants living in the United States (*n* = 407) in 49 states and territories were recruited through this online national study and completed surveys of their ageist beliefs, epistemological style, social dominance orientation (SDO), and right-wing authoritarianism (RWA). RWA, SDO, and naïve realism were all positively associated with ageist beliefs. A hypothesized path model and two alternative models suggested the retention of a model whereby naïve realism led to RWA, which led to SDO, and finally to ageism. All possible direct and indirect effects were significant within the retained model, suggesting the presence of a multiple mediation. The fit of this model was superior to that of models testing alternative theoretical causal chains. Naïve realism may lead to authoritarian aggression, authoritarian submission, and conventionalism, which may then increase the value that people place on social hierarchies, and this may influence the development and retention of ageist beliefs. Helping people to understand what their basic beliefs about the world are and how they may play a role in the development of ageism may assist in reducing ageist attitudes.

## 1. Introduction

Ageism is a system of stereotypes, prejudices, and discrimination that older adults experience because of their age and the process of ageing [1, 2]. At its most basic, ageism is the prejudice of one group of individuals towards another group of individuals due to age [2]. People of any age may be stereotyped or experience discrimination because of their age, but it is generally thought of as a systematic result of advancing chronological age [1, 3]. Ageism has been further defined as having three forms: (a) limitations of opportunity or activities based on positive or negative age-based stereotypes; (b) a culturally held belief that one's social position, psychological characteristics, or individual experience are in part defined by their age; or (c) age being always a germane variable to study and/or that the results of one age-group can be generalized to another or all age-groups [3]. These three forms of ageism begin to suggest the numerous ways in which ageism can pervade the society. Due to the pervasive nature of ageism and the profound negative effects it can have on older adults, the current study seeks to understand the factors which contribute to individuals developing and holding ageist beliefs. Dual process theory is used to examine how one's worldview, beliefs in social hierarchy, authoritarian aggression, authoritarian submission, and conventionalism predict ageism.

As a form of prejudice, ageism—like other forms of prejudice—has both short- and long-term consequences [4]. Aging has two important components: the biological process of aging and the social construction of aging [3]. Age is the only social identity with subcategories that everyone has the possibility of joining [4]. As a result, an overwhelming number of people will experience age discrimination, with estimates as high as 89% in previous studies [1]. In a study of 28 European countries, age was the most frequently mentioned reason for discrimination [5]. Defining the boundary of old age has also become more challenging, as the population ages and life expectancy increases [4, 6–9].

Ageist stereotypes and prejudice can be cultivated across the life span. People may internalize stereotypes about age when they are younger, which may become more salient to them as they age [8]. Individuals with less exposure to older adults when they are younger or middle aged may have more general or negative perceptions of aging and the stereotypical declines that accompany aging, which leave these individuals and older adults vulnerable to age-based discrimination and prejudice [6, 8]. As a result of these stereotypes and prejudices, younger individuals may attempt to create psychological and physical distance from older adults [10]. Even other older adults may not associate themselves with old age, despite being seen that way by others [10]. All of this contributes to the wide range of areas in which ageism may impact a person's life.

Ageism takes a number of forms and occurs across many domains of life. Broadly, this includes employment [11, 12], healthcare [1, 3], research [9, 13], overaccommodation [14], elder abuse [14], media [4], and everyday life and conversation [3]. Ageism in these areas influences individuals' attitudes, cognitions, and behaviors toward older adults [1]. This has the potential to negatively affect social and economic opportunities [5, 9], physical functioning [6, 9], cognitive and mental health [5, 6, 9], satisfaction with life [5], and overall quality of life of older adults [15]. These broad domains can have very profound, specific impacts on elderly individuals' lives.

Researchers have identified some specific negative impacts of ageism. Studies have shown exposure to ageist stereotypes has affected handwriting [1, 16], balance [16], gait speed [16], memory [1, 6], self-confidence [1], hearing [6], self-care [16], and cardiovascular event risk and recovery time [6, 16]. For older adults, repeated occurrences of ageist stereotypes have the potential of becoming a chronic stressor [1]. When ageism becomes a chronic stressor, it has been linked to negative mental health outcomes, hypertension, adverse birth outcomes, obesity, and heart disease [1].

Dual process theory addresses how two different processes can produce a thought, or, in other words, how a thought can arise in two different ways. It has been suggested using dual process theory that individual prejudice is the result of two motivational goals. These motivations are the dominance-power-superiority motivation and the threat-driven group defense and social control motivation [17]. Dual process theory has been used as a model to link ideological attitudes, social worldviews, personality, and intergroup attitudes to various forms of prejudice [17–19]. Duckitt [17] created a causal model linking worldview to right-wing authoritarianism, social dominance orientation, and then to in- and out-group attitudes. The current study uses this same framework linking epistemological style (worldview), right-wing authoritarianism, social dominance orientation, and ageism.

Naïve realism is an epistemological style, meaning that it is a set of beliefs about what knowledge is and how it is acquired [20]. There are many different epistemological styles: realism, romanticism, constructionism, empiricism, rationalism, metaphorism, pragmatism, dualism, relativism, positivism, humanism, emotionalism, transcendentalism, individualism, etc. [20]. These are all different attempts to understand knowledge beliefs [20]. The reason epistemological style is important is because there are consequences to maintaining such a worldview [20]. It is important to pay attention to how events are being understood and judged by individuals, as these might provide clues about how individuals perceive and interpret the world around them [21]. Naïve realism is a personal epistemology which may serve as a means by which past events may impact current judgements [22]. Naïve realism is characterized by a belief that knowledge is composed of facts, doubts are frustrating, and there is correct answer for every question [20]. This style is characterized by a “what you see is what you get” type of thinking that is not interested in contextualization [20]. It has been argued that one's epistemological style contributes to the development and maintenance of prejudicial beliefs, although to date there is little evidence directly linking epistemological style and prejudice [23, 24]. However, epistemological style has been linked to both social dominance orientation and right-wing authoritarianism, which have been linked to prejudice [19]. Additionally, other forms of worldview, such as dangerous world and competitive world jungle, have been associated with prejudice, supporting the importance of including worldview factors [17]. The tenets of naïve realism have been empirically demonstrated in past research, suggesting the practical importance of understanding how naïve realism operates as a worldview factor [21, 25–28].

Social dominance orientation (SDO) is the value individuals place on the hierarchy structure of relationships between social groups [29, 30]. Social dominance is thought to be a stable trait and has been linked to the development prejudice [29]. There are over two decades of research linking SDO to generalized prejudice, both discriminatory behaviors and individual levels of prejudice [29]. In one study, it was found that individuals who had high SDO scores used prejudice to affirm or uphold the dominance of their in-group [30]. People with high SDO are the most resistant to changing prejudicial attitudes because it helps maintain the social hierarchy [30]. Even controlling for personality traits and cognitive style, SDO has been positively correlated with racial prejudice, homosexual prejudice, ethnocentrism, racial superiority, anti-Arab racism, modern racism, and sexism [31].

Right-wing authoritarianism (RWA) is composed of three attitudinal clusters: authoritarian aggression, authoritarian submission, and conventionalism [32]. Authoritarian aggression is the process by which established authorities create legitimized, generalized targets for aggression of out-groups and perceived deviants [33]. Authoritarian submission is deference to the perceived legitimate authorities of one's society [33]. Conventionalism is strict adherence to the norms and conventions of one's society that are endorsed by the authority and the conviction that everyone else should comply as well [33]. RWA has been linked to various types of prejudice and bias [32, 34], including ethnocentrism, racism, homosexual prejudice, and sexism [31, 32]. It has also been correlated with prejudice against each of the following groups: aboriginal peoples, Africans, AIDS patients, Arabs, Blacks, Chinese, feminists, Filipinos, Hispanics, Japanese, Jews, Pakistanis, and Sikhs [31, 34].

Even though RWA and SDO have both been correlated with many similar forms of prejudice, research has shown that they are separate ideological components [18]. RWA and SDO have been linked to different beliefs about the nature of the world (i.e., if it is a dangerous place) and correlated with different personality traits [18]. This suggests that, although RWA and SDO are both related to prejudice, the explanations are different [18]. For example, a person high in SDO may use prejudice to reinforce the status of their social group and maintain the hierarchical order [18]. On the other hand, someone high in RWA may see an out-group member as either a target (authoritarian aggression) or threat to social norms (conventionalism). Through these different mechanisms, RWA and SDO are thought to mediate the relationship between worldview and prejudice [35, 36].

Although research has generally examined the constructs of naïve realism, RWA, SDO, individually and linked them to generalized prejudice, there is limited research specifically linking these concepts in a theoretical chain with ageism. As a result, the purpose of the current study is to integrate these constructs into a path model with data from a national sample of U.S. adults. Using the dual process model framework and previous research [17], it is hypothesized that the association between naïve realism and ageism will be simultaneously mediated by RWA and SDO, such that higher levels of naïve realism will be associated with higher levels of RWA and SDO and, in turn, higher levels of ageism.

## 2. Materials and Methods

### 2.1. Participants

Participants living in the United States (initial *n* = 416) were recruited online via Amazon's Mechanical Turk (Mturk; http://www.mturk.com). Mturk tracks the number of times a participant completes a survey, and this revealed seven individuals took the survey twice, resulting in the second set of their data being removed. With these exclusions, this left a total of 409 participants. Of the remaining 409 participants, two did not respond correctly to all seven of the random attention check questions in the survey (ACQs; e.g., “Please select “Strongly agree” for this item”). The data from those two individuals were also removed. The use of ACQs on Mturk has been demonstrated to help improve the quality of data that can be obtained [37]. This left a total sample size of 407 participants representing 49 of the U.S. states and territories.

Participants ranged in age from 18 to 77 years (*M* = 36.72; SD = 12.75), with self-reported political affiliation: 44.5% Democrat, 31.7% Independent, 16.5% Republican, 4.4% Libertarian, 2.0% Green Party, and 1.0% Tea Party. The majority of the sample (87.5%) identified as heterosexual, with 5.7% bisexual, 4.4% gay or lesbian, and 2.5% queer. Participants further identified as transgender/nonbinary (*n* = 8), male (*n* = 164) and female (*n* = 235). The race/ethnicity of the participants was as follows: 77.4% white/European American (non-Latino), 7.4% Asian/Asian American/Pacific Islander, 6.1% black/African-American (non-Latino), 5.2% Latino/Hispanic, 3.4% multiracial/multiethnic, and 0.5% American Indian/Native American. Most of the participants in the sample also had some education beyond high school: 0.5% had only finished grade school, 8.6% had a high school diploma or GED, 9.6% completed a 2-year/technical school degree, 20.9% had completed some college (no degree), 43.2% had a 4-year college degree, 14.5% held a master's degree, and 2.7% reported a doctorate degree.

### 2.2. Measures

#### 2.2.1. Epistemological Style Inventory (ESI)

The 15-item ESI assesses one's epistemological orientation [20]. Of the three subscales (naïve realism, logical inquiry, and skeptical subjectivism), only the naïve realism subscale was used in the current study due to its theoretical link to the other constructs in the study. While the original scale authors did not report the ESI's internal consistency or that of any of the subscales, the present study demonstrates acceptable reliability of the naïve realism subscale at *α* = 0.73.

#### 2.2.2. Social Dominance Orientation Scale (SDOS)

The 16-item SDOS examines one's desire for a social order in which one's in-group is dominant over out-groups, and for hierarchy-enhancing policies and ideologies [38]. This scale has demonstrated high levels of internal consistency (*α* = 0.91) [38]. In this study, the SDOS had strong internal consistency at *α* = 0.95.

#### 2.2.3. Short Version of the Right-Wing Authoritarianism (RWA) Scale

Right-wing authoritarianism was measured by the 15-item short version of the RWA scale [39]. This scale assesses three primary characteristics of RWA: authoritarian aggression, authoritarian submission, and conventionalism, although only the total score was used in the current study. This scale has demonstrated acceptable internal consistency (*α* = 0.72) [39]. In the current study, the scale had strong internal consistency at *α* = 0.92.

#### 2.2.4. Intolerant Schema Measure (ISM)

The ageism subscale of the Intolerant Schema Measure was used to assess ageist ideology [40]. The ageism subscale contains nine items and has shown acceptable internal consistency (*α* = 78) [40]. In the current study, the ageism subscale showed good internal consistency at *α* = 0.81.

### 2.3. Procedure

Participants were recruited through Mturk to complete an online survey. The Institutional Review Board at the host university approved the study prior to beginning recruitment. As a platform, Mturk allows the recruitment of participants to complete human intelligence tasks (HITS), such as online self-report surveys similar to the one used in the current study. On Mturk, no identifying information (e.g., names and social security numbers) is allowed to be collected, so the study was anonymous. Participants (known as workers on Mturk) get to select which HITs to participate in for compensation. After picking a HIT, participants are given the instructions and a preview of the task. Once the HIT is completed, the participant is compensated by the researcher. Participants were paid $1 for completing the current study.

### 2.4. Data Analysis Plan

Tests of normality (skewness and kurtosis) were run to determine whether the variables are normally distributed. A correlation matrix was generated to show the bivariate relationships between the variables in the path analysis. A path model was then constructed in AMOS 22 [41], reflecting the theoretical pathways hypothesized. Variables in the model included naïve realism, SDO, RWA, and ageism. Direct effects were examined between all variables (directional arrows in the path model), as well as indirect (mediational) effects from naïve realism to ageism. The following criteria were used to assess goodness of fit of the model: ratio of chi-square to degrees of freedom less than 2.0; fit indices, including the comparative fit index (CFI), goodness of fit index (GFI), adjusted goodness of fit index (AGFI), normed fit index (NFI), incremental fit index (IFI), and Tucker-Lewis index (TLI), higher than 0.90 to indicate adequate fit, and greater than 0.95 to indicate good fit [42–44]; and a root mean square error of approximation (RMSEA) of 0.08 or less to indicate adequate fit, and 0.05 or less to indicate good fit [44, 45].

## 3. Results

### 3.1. Data Screening and Preliminary Analyses

The data were screened for univariate outliers. The SDO had three outliers, and ageism subscale had four. As this accounted for less than 2% of the data, these were retained [46]. No multivariate outliers were detected (*D*^2^ > 16.27). Data were also checked for normality via skewness and kurtosis. All values fell below the ±2.0 cutoff (skewness −0.05 to 1.30; kurtosis −0.75 to 1.89).

### 3.2. Bivariate Correlations

A correlation matrix was calculated showing all of the bivariate relationships among variables in the current study (Table 1). All factors of the hypothesized model were significantly and positively associated.

### 3.3. Path Analysis

**Figure 1:**
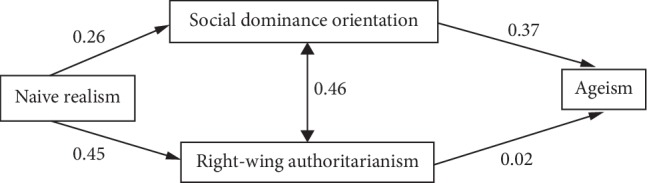
Standardized regression weights of hypothesized model.

A path model was developed to evaluate a hypothesized pattern of relationships among variables, leading from naïve realism simultaneously through SDO and RWA to ageism. This model further specified an indirect effect of naïve realism on ageism simultaneously through RWA and SDO. The hypothesized path model with factor loadings (standard regression weights) appears in Figure 1. All paths were statistically significant (all *ps* < 0.001), except for the path between RWA and ageism, which was not (*p*=0.704). The model explained 14% of the variance in ageism, a medium-size effect [47]. The overall fit of the model had a mix of good, adequate, and below adequate indicators (Table 2) that taken together suggested the model had adequate fit. With the only adequate fit and the nonsignificant path loading between RWA and ageism, two alternative models were tested.

**Figure 2:**
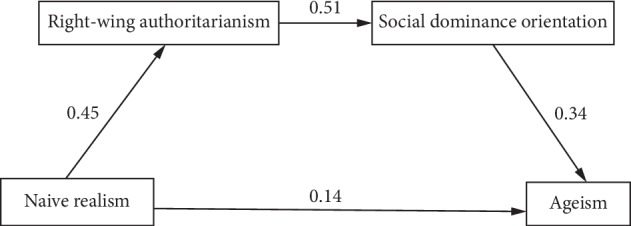
Standardized regression weights of first alternative model with naïve realism leading to RWA to SDO followed by ageism.

The two alternative models were indicated, both by the results of the first model and supported by studies which indicate the differential effects of RWA and SDO [48]. The first alternative model specified that naïve realism would have a direct effect on RWA, which in turn would have a direct effect on SDO, and finally SDO would have a direct effect on ageism. This model further specified an indirect effect of naïve realism on SDO through RWA, of naïve realism on ageism through RWA and SDO, and RWA on ageism through SDO. The first alternative path model with factor loadings (standard regression weights) appears in Figure 2. The model explained 16% of the variance in ageism, a medium-size effect [47]. The overall fit of the model was good (Table 2).

**Figure 3:**
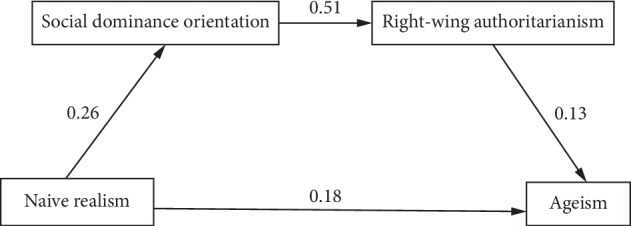
Standardized regression weights of first alternative model with naïve realism leading to SDO to RWA followed by ageism.

The second alternative model specified that naïve realism would have a direct effect on SDO, which in turn would have a direct effect on RWA, and finally RWA would have a direct effect on ageism. This model reverses RWA and SDO from the first alternative model to examine potential reverse causal effects of the moderation [49, 50]. This model further specified an indirect effect of naïve realism on RWA through SDO, of naïve realism on ageism through SDO and RWA, and SDO on ageism through RWA. The second alternative path model with factor loadings (standard regression weights) appears in Figure 3. The model explained 5% of the variance in ageism, a small-size effect [47]. The overall fit of the model failed to achieve adequate levels (Table 2). Therefore, the first alternative model was retained.

For the retained model, alternative model 1, naïve realism was positively associated with RWA, RWA was positively associated with SDO, and SDO was positively associated with ageism (all *ps* < 0.001). Additionally, naïve realism yielded a significant indirect effect on SDO through RWA (*β* = 0.23, *p* < 0.001), as well as a significant indirect effect on ageism through RWA and SDO (*β* = 0.08, *p* < 0.001), and RWA yielded a significant indirect effect on ageism through SDO (*β* = 0.18, *p* < 0.001). All possible indirect effects and all betas were greater than or equal to 0.08, and all *p*'s less than or equal to 0.001, suggesting that every possible mediation, including multiple mediation effects, was statistically significant.

## 4. Discussion

This study examined the relationships between psychological factors that may contribute to the development and endorsement of ageist attitudes. Dual process theory was used to examine how epistemological style, SDO, and RWA predict ageist attitudes. The hypothesized model (Figure 1) was that the association between naïve realism and ageism would be simultaneously mediated by RWA and SDO, such that higher levels of naïve realism would be associated with higher levels of RWA and SDO and, in turn, higher levels of ageism. The path model demonstrated only adequate model fit. Two alternative models were tested, with alternative model one demonstrating good model fit and alternative model two (Figure 3) failing to achieve adequate model fit. Therefore, the hypothesis was only partially supported and alternative model one was retained. The retained model path went from naïve realism to RWA, which led to SDO, and finally ageism (Figure 2; containing coefficients for the retained model). All direct and indirect effects were statistically significant.

The positive relationship between naïve realism and RWA is similar to previous research [17] in that epistemological styles had small-to-medium positive direct effects on RWA. A positive relationship between RWA and SDO supports the strong and robust connection found in the literature between RWA and SDO [17, 18]; however, the directional path from RWA to SDO, in comparison to the reverse path tested in alternative model two, has not yet emerged in the extant literature. One reason for this might be because ageism operates differently from other forms of prejudice [51–53]. This may be for a few reasons including that age is the only social identity that everyone has the possibility of joining [4], people may cultivate ageist beliefs across a life span—internalizing them when they are younger and becoming more salient as they age [8], and ageism has been posited as a protective or defensive mechanism [54, 55]. Finally, there was a positive relationship between SDO and ageism, which is the first time to the authors' knowledge that this effect has emerged in the literature. In addition to the direct effects, there was a significant indirect effect from naïve realism to ageism through both RWA and SDO. This suggests that naïve realism may lead to authoritarian aggression, authoritarian submission, and conventionalism, which may then increase the value that people place on social hierarchies, and this may influence the development and retention of ageist beliefs.

### 4.1. Implications

Understanding the relationship between naïve realism, RWA, SDO, and ageism may illuminate the mechanisms behind why people may form or hold ageists beliefs. The relationships among naïve realism, RWA, and SDO may function at the core of a person's worldview and underlie their belief systems. Ageist attitudes and beliefs are likely manifestations of this worldview. It is possible that in order to make changes to ageist attitudes, focusing on the underlying structure is important [35]. Helping people to understand what their basic beliefs about the world are and how they may play a role in the development of ageism may help reduce ageist attitudes—particularly interventions which prompt questioning of social hierarchies or which draw upon social norms via intergroup contact with older adults [56]. However, much more research in this area is necessary before any concrete recommendations can be made.

In the context of healthcare, it is well documented that up-and-coming healthcare providers—from all disciplines—are opting out of gerontological electives and rotations during their training, despite the increasing need for care [57, 58]. Interventions for medical students such as the Aging Game, the Road of Life, and Half-Full Aging Simulation Experience have shown mixed results at attitudinal changes [58]. These interventions are all simulation-based learning activities to help students understand the aging process [58]. Health-care advocates and educators suggest that not only do medical students need geriatric content in the curriculum, but positive experiences with older adults as well [58]. Given the mixed results for the simulation-based interventions, learning about the mechanisms behind ageism may allow for more targeted intervention to reduce the high level of ageist prejudice and discrimination that exists. If older adults have fewer experiences of ageist discrimination, it will hopefully help reduce the negative impacts of ageism.

### 4.2. Limitations and Future Directions

This study has several limitations, which suggest areas for future research. The Mturk sample was generally more progressive and well educated than the U.S. population. It would be beneficial in future research on the underpinnings of ageism to use alternative recruitment strategies, such as in-person through community centers, low-income clinics, and in rural areas, in order to recruit a more diverse sample. Another limitation is that the data were cross-sectional. While these results suggest the retention of theoretical causal model over other causal models, causality was not proven. There is the potential for bidirectionality, in which ageist attitudes might influence SDO, RWA, and epistemology. However, the lack of support for the alternative models makes this less likely. Future experimental research should examine whether one way to change ageist beliefs is via change in these other variables. A final limitation was the epistemological measure. The naïve realism subscale *α* (0.73) is only fair for a subscale. Ideally, for a scale, 0.80 is considered good. This suggests there may have been some error introduced in the measurement of this construct and, as a result, the measure may not have been associated with other measures to the extent that a stronger scale could illuminate.

Future research may also want to investigate factors which reduce ageist beliefs or predict positive attitudes about aging, as opposed to the negative form of ageism measured in this study. Negative ageism is the more commonly studied form of ageism and focuses on attitudes towards older adults and the process of aging, discrimination toward older adults, and policies and practices which may perpetuate stereotypes about older adults [9]. Positive ageism, on the other hand, focuses on the strengths of the aging process and older adults.

## 5. Conclusions

This study assessed psychological factors that may contribute to the development and endorsement of ageist belief systems. The results suggest the retention of a path model whereby naïve realism led to RWA, which led to SDO, and finally to ageism. This indicated naïve realism may lead to authoritarian aggression, authoritarian submission, and conventionalism, which may then increase the value that people place on social hierarchies, and this may influence the development and retention of ageist beliefs. Helping people to understand what their basic beliefs about the world are and how they may play a role in the development of ageism may assist in reducing ageist attitudes.

## Figures and Tables

**Table 1:** Bivariate correlations.

Correlations				
	1	2	3	4
1 Ageism				
2 RWA	0.209^*∗∗*^			
3 SDO	0.378^*∗∗*^	0.514^*∗∗*^		
4 Naïve realism	0.233^*∗∗*^	0.449^*∗∗*^	0.263^*∗∗*^	

^*∗∗*^ = Correlation is significant at the *p* < 0.01 level (2-tailed).

**Table 2:** Model fit indices for hypothesized and alternative models.

Fit Indices	Model 1	Model 2	Model 3
Chi-square ratio	9.70	1.42^*∗*^	53.37
Comparative fit index (CFI)	0.969^*∗∗*^	1.00^*∗∗*^	0.63
Goodness of fit index (GFI)	0.988^*∗∗*^	0.998^*∗∗*^	0.888
Adjusted goodness of fit index (AGFI)	0.883	0.991^*∗∗*^	0.442
Normed fit index (NFI)	0.966^*∗∗*^	0.995^*∗∗*^	0.631
Incremental fit index (IFI)	0.97^*∗∗*^	1.00^*∗∗*^	0.635
Tucker-Lewis index (TLI)	0.815	1.00^*∗∗*^	−0.111
Root mean square error of approximation (RMSEA)	0.146	<0.001	0.359

Note that ^*∗*^is adequate fit (>0.90 for all, except RMSEA <0.08) and ^*∗∗*^is good fit (>0.95 for all, except RMSEA <0.05); ^*∗*^chi-square ratio <2.0.

## Data Availability

Readers can access the data supporting the conclusions of the study by contacting the corresponding author.
